# Characterization and phylogenetic analysis of the complete mitochondrial genome of *Aipysurus eydouxii* Gray 1849 (Elapidae: Hydrophiinae)

**DOI:** 10.1080/23802359.2024.2419423

**Published:** 2024-10-25

**Authors:** Shiying Wei, Xiaowan Ma, Hongtao Liu, Yan Xu, Longyan Zhao, Guoqiang Huang, Lianghua Huang, Ying Qiao, Shengping Zhong

**Affiliations:** a Institute of Marine Drugs, Guangxi Key Laboratory of Marine Drugs, Guangxi University of Chinese Medicine, Nanning, China; b Key Laboratory of Tropical Marine Ecosystem and Bioresource, Fourth Institute of Oceanography, Ministry of Natural Resources, Beihai, China; c Hainan Provincial Key Laboratory of Tropical Maricultural Technologies, Haikou, China; d Guangxi Beibu Gulf Marine Research Center, Guangxi Academy of Sciences, Nanning, China; e Guangxi Engineering Technology Research Center for Marine Aquaculture, Guangxi Institute of Oceanology Co., Ltd., Beihai, China

**Keywords:** Mitochondrial genome, *Aipysurus eydouxii*, Hydrophiinae

## Abstract

Hydrophiine sea snakes represent ecologically significant and species-rich marine predatory reptiles, many of which inhabit marine environments throughout their entire lifecycles. However, due to morphological variability and limited molecular phylogenetic studies, the taxonomic relationships within this group remain unclear. In this study, we present the first complete mitochondrial genome of Aipysurus sea snakes, specifically *Aipysurus eydouxii* Gray 1849. The mitogenome comprises 17,228 base pairs and contains a total of 37 genes , plus a putative control region. This study provides valuable genetic data that will contribute to the future taxonomic classification and ecological protection of hydrophiine sea snakes.

## Introduction

The true sea snakes (Hydrophiinae), or hydrophiine sea snakes, are viviparous predatory reptiles, which are the largest species diversity group of extant marine benthic reptiles (Lukoschek 2007; Xiaokaiti et al. 2022). The hydrophiine sea snakes currently contain more than 61 living species and mostly inhabit the shallow waters or inshore habitats throughout the Indo-West Pacific (Sanders et al. 2012), many of which are inhabited in northern Australia and South East Asia (Yi et al. 2020). The two species-rich groups of hydrophiine sea snakes are the Hydrophis and Aipysurus sea snakes (Kim et al. 2020). The Aipysurus sea snakes contain 11 species in two genera: *Aipysurus* and *Emydocephalus* (Lukoschek 2018). The monophyletic relationship of Aipysurus group has been resolved based on morphological and molecular data. However, due to difference in accurate identification and classification of taxonomic characteristics within Aipysurus group, synonymy and misidentification of Aipysurus species have been reported recently (Sanders et al. 2012). *Aipysurus eydouxii* Gray 1849, also known as the marbled sea snake, is particularly unique Aipysurus species because it not only has the most widely distributed habitat from Asia to Australia but it also has secondary reduction of fangs and venom glands for its ecological change (Li et al. 2005). An allopatric species from Australia and New Guinea within the *A. eydouxii* population has been reported, and molecular data revealed significant divergence between the Australian and Asian populations. However, the complete mitochondrial genome of *Aipysurus* species has yet to be revealed. Here, we report the first complete mitochondrial genome of *A. eydouxii*, which will afford useful molecular data for classification and phylogenetic analyses in Aipysurus sea snakes.

## Materials

**Figure 1.**
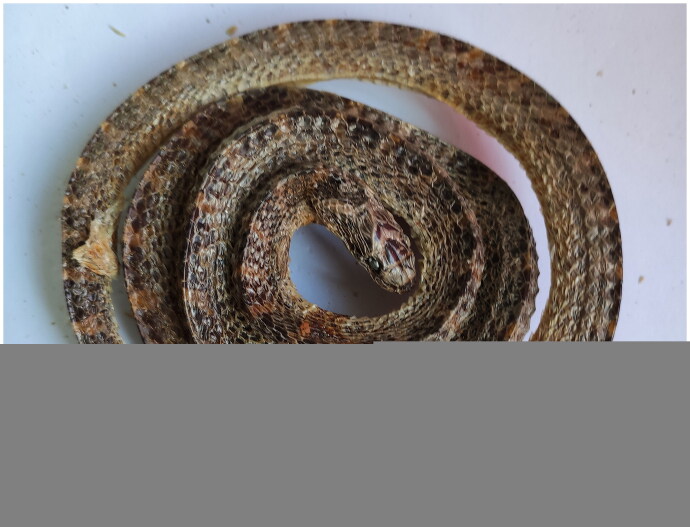
Photograph of morphology features of the marbled sea snake *Aipysurus eydouxii* Gray 1849. The photos of *Aipysurus eydouxii* were taken at Marine Biological Museum, Guangxi Institute of Oceanology, Beihai, China (photographed by Shengping Zhong). The species was identified using scalation and color pattern as the classic characteristics.

A dried dead specimen of *A. eydouxii* was collected from a local dried seafood market in Hai Nan province, China (QiongHai, 19.273288 N, 110.497589 E) and the whole body specimen (#HS0106) was deposited at Marine Biological Museum, Guangxi Institute of Oceanology, Beihai, China (http://www.gxas.cn/kypt/kxpj/kpcg, Shengping Zhong, shpzhong@foxmail.com) (Figure 1).

## Methods

The total genomic DNA was extracted from the muscle of the specimen using an SQ Tissue DNA Kit (OMEGA, Guangzhou, China) following the manufacturer’s protocol. DNA libraries (300 bp insert) were constructed with the TruSeq Nano™ kit (Illumina, San Diego, CA) and were sequenced (2 × 150 bp paired-end) using HiSeq platform at BGI Company (Shenzhen, China). Mitogenome assembly was performed with MITObim (Hahn et al. 2013). The cytochrome b (cytb) gene of *A. eydouxii* (GenBank accession number: JX423395) (Sanders et al. 2012) was chosen as the initial reference sequence for MITObim (v.1.9.1) assembly. Gene annotation was performed by MITOS (v.1.1.1) (http://mitos.bioinf.uni-leipzig.de/index.py). For phylogenetic analysis of true sea snakes, the relevant mitochondrial genomes of 13 species from Elapidae were retrieved from GenBank. Coding sequences of 13 genes that shared by all taxa were extracted into a continuous super DNA sequences in the same order. The phylogenetic tree was constructed from the Coding DNA sequences combined in the same order. The MAFFT (v. 7.520) software (Katoh and Standley 2013) was applied to align the super DNA sequences used L-INS-I algorithm. The maximum-likelihood phylogenetic tree was constructed using the PhyML online web service (http://www.atgc-montpellier.fr/phyml/, v3.3.20220408) with Smart Model Selection. The number of bootstrap replicates was 1000. The circular mitogenome map was visualized using OGView online tool (http://www.1kmpg.cn/ogview).

## Results

### Basic features of the *A. eydouxii* mitogenome

**Figure 2.**
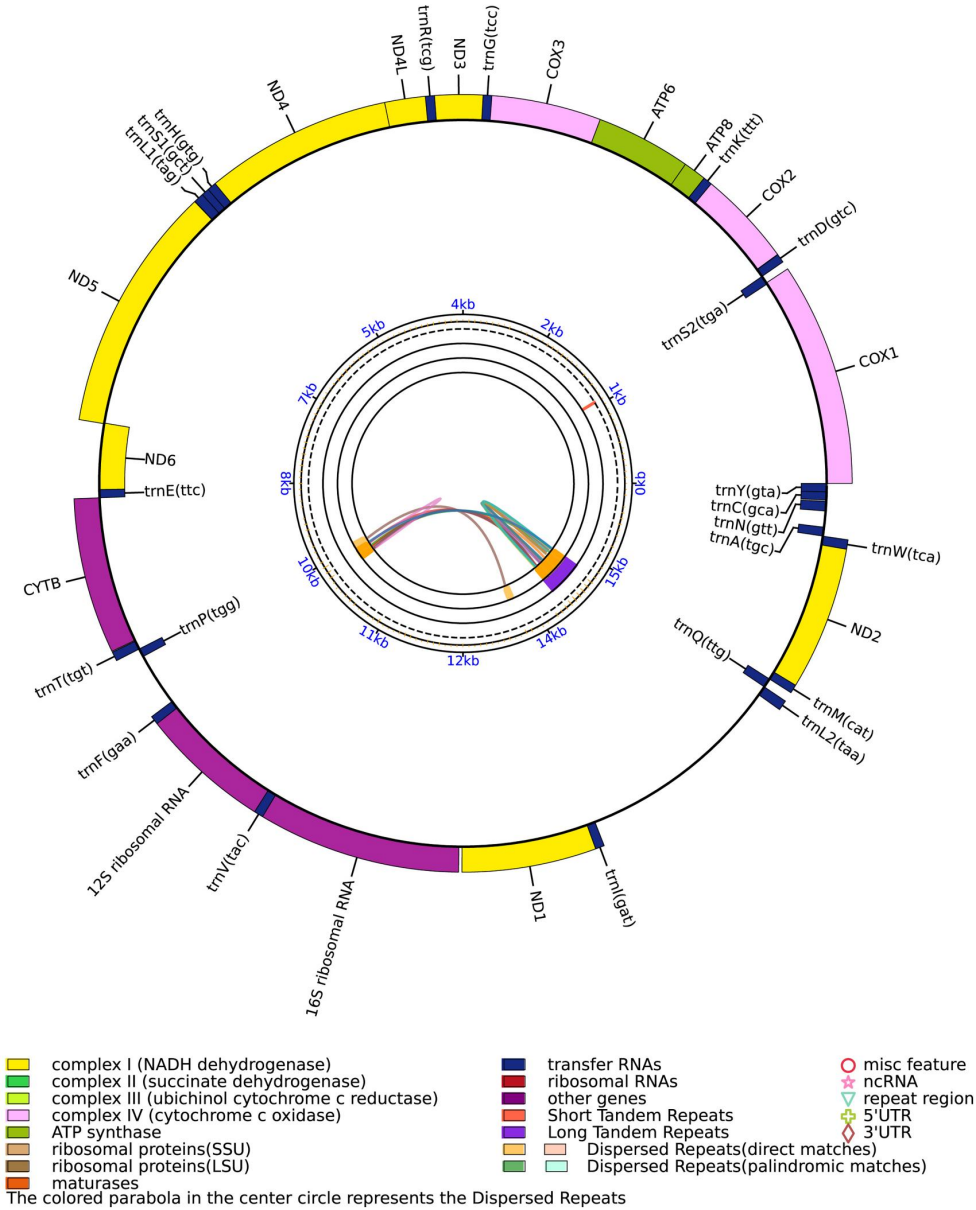
Gene map of the complete mitochondrial genome of *A. eydouxii.* The complete mitochondrial genome of *A. eydouxii* contains a total of 37 genes including 13 protein-coding genes, two ribosomal RNA genes, and 22 transfer RNA genes.

The entire mitogenome of *A. eydouxii* (GenBank accession number: MZ242140) is 17,228 bp long and contains a conserved collection of 13 protein-coding genes (PCGs), two ribosomal RNA genes, 22 transfer RNA genes, and a putative control region (Figure 2). A total of 37 genes were annotated, with 725 nucleotides identified as putative control region. The result of the repetitive sequence identification showed the control region of the mitogenome of *A. eydouxii* contains tandem repeat sequences (Figure 2). The sequencing depth and coverage map were calculated and shown in Supplementary Figure 1 (maximum coverage 8095×, minimal coverage 59×, and average coverage 450×). The result of the OGView showed no cis-splicing and trans-splicing genes in the mitochondrial genome of *A. eydouxii*. The overall base composition of the mitogenome is predicted to be A 33.47%, T 26.45%, C 27.37%, and G 12.71%, with a somewhat higher A + T content of 59.92%, which is similar to, but slightly lower than, *Emydocephalus ijimae* (60.7%) (Yi et al. 2019) within Aipysurus sea snakes.

### Phylogenetic analysis

**Figure 3.**
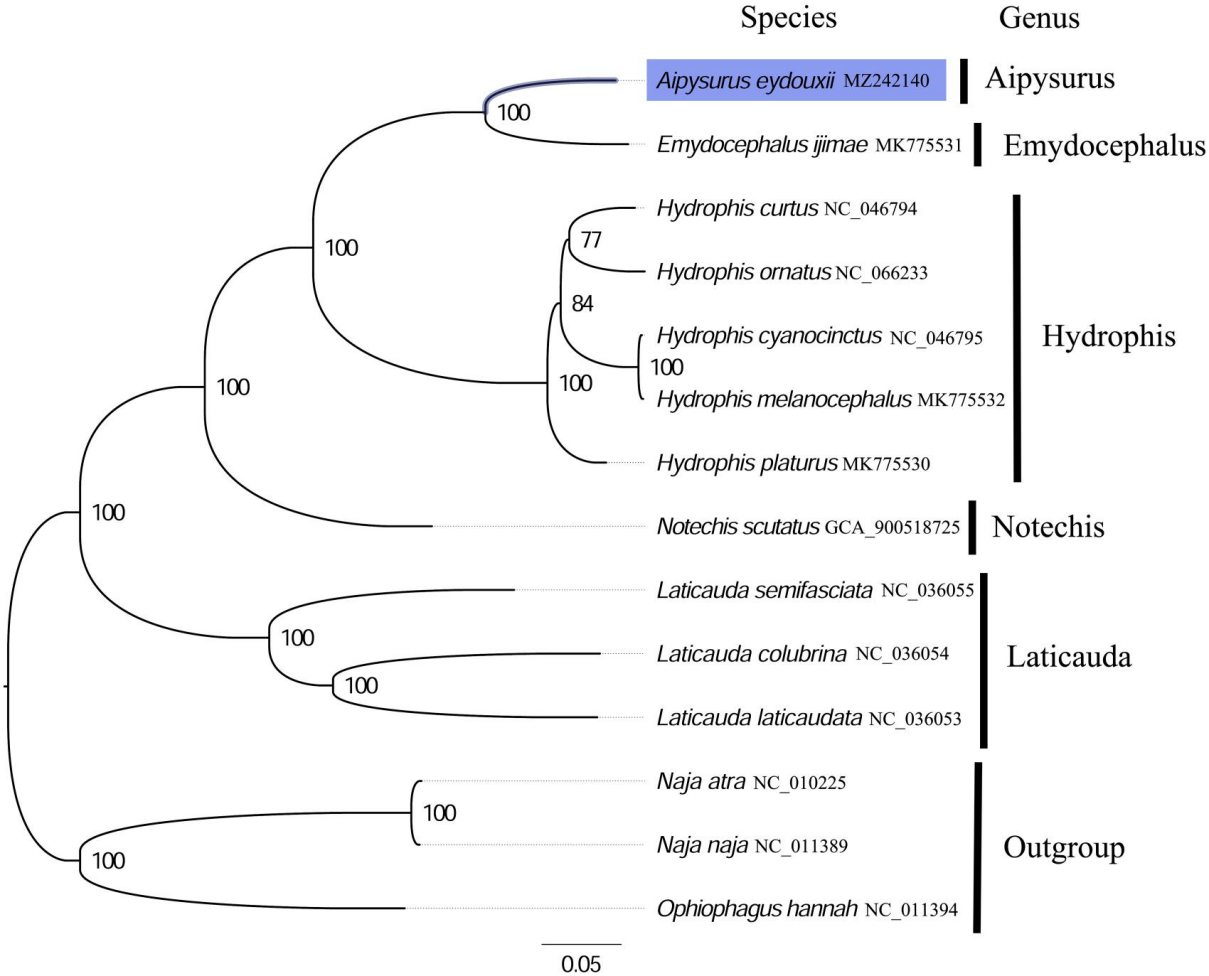
Phylogenetic tree of 13 species in Elapidae. The complete mitogenomes were downloaded from GenBank and the following sequences were used *Laticauda colubrina* NC_036054 (Kim et al. 2018), *L. laticaudata* NC_036053 (Kim et al. 2018), *L. semifasciata* NC_036055 (Kim et al. 2018), *Emydocephalus ijimae* MK775531 (Yi et al. 2019), *Hydrophis platurus* MK775530 (Yi et al. 2019), *H. melanocephalus* MK775532 (Yi et al. 2019), *H. curtus* NC_046794 (Qiu et al. 2019), *H. cyanocinctus* NC_046795 (Qiu et al. 2019), *H. ornatus* NC_066233 (Xiaokaiti et al. 2022), *Naja atra* NC_011389 (unpublished), *N. naja* NC_010225 (Yan et al. 2008), *Notechis scutatus* GCA_900518725 (unpublished), and *Ophiophagus hannah* NC_011394 (Chen and Lai 2010). The phylogenic tree based on the concatenated nucleotide sequences of 13 mitochondrial PCGs was constructed by maximum-likelihood method via PhyML online server (http://www.atgc-montpellier.fr/phyml/), using GTR substitution model with 1000 bootstrap replicates. The bootstrap values are indicated at each branch nodes; terrestrial elapids were rooted to be outgroup species.

The phylogenetic analysis inferred from the concatenated nucleotides sequences of 13 PCGs suggests that *Aipysurus* and *Emydocephalus* sea snakes have close relationship (Figure 3). Aipysurus and Emydocephalus sea snakes initially constituted Aipysurus group clade, which subsequently clustered with the Hydrophis sea snakes clade to form the Hydrophiine sea snakes group (Figure 3). These results are consistent with the phylogenetic analyses of hydrophiine sea snakes based on mitochondrial and nuclear sequences (Sanders et al. 2013). Moreover, the mainland tiger snake (*Notechis scutatus*) was also revealed to be more phylogenetic closer to hydrophiine sea snakes group than sea krait group, which is consistent with the phylogenetic analyses of Elapidae snakes based on whole genome sequences (Ludington and Sanders 2021).

## Discussion and conclusions

By employing high-throughput sequencing technology, we successfully obtained and annotated the complete mitochondrial genome of the marbled sea snake, *A. eydouxii*. Our findings reveal that the gene order and composition in *A. eydouxii* are identical to those observed in the typical mitogenomes of other sea snakes, corroborating previous reports (Xiaokaiti et al. 2022). Furthermore, the phylogenetic analysis conducted in this study, which integrated the concatenated nucleotides sequences of 13 PCGs of mitogenome, demonstrated that the relationships within the Hydrophiine sea snake group are consistent with earlier studies based on morphological and molecular data (Lukoschek 2007; Sanders et al. 2013). Importantly, the complete mitochondrial genome sequence of *A. eydouxii* represents the first fully sequenced mitogenome within the Aipysurus sea snakes. This novel genomic data provides a valuable resource for future studies aimed at resolving ongoing classification and phylogenetic controversies within the hydrophiine sea snakes.

## Supplementary Material

Supplemental Material

OGV result showed no complex genes.html

## Data Availability

The genome sequence data that support the findings of this study are openly available in GenBank of NCBI at https://www.ncbi.nlm.nih.gov/ under the accession no. MZ242140. The associated BioProject, SRA, and Bio-Sample numbers are PRJNA823815, SRR18651219, and SAMN27361759, respectively.
