# Supplementary figures and images for: Characterization and phylogenetic analysis of the complete mitochondrial genome of Aipysurus eydouxii Gray 1849 (Elapidae: Hydrophiinae)
Source: Mitochondrial DNA B Resour. 2024 Oct 25;9(10):1450–4. doi: 10.1080/23802359.2024.2419423 (PMC11514406; doi:10.1080/23802359.2024.2419423)

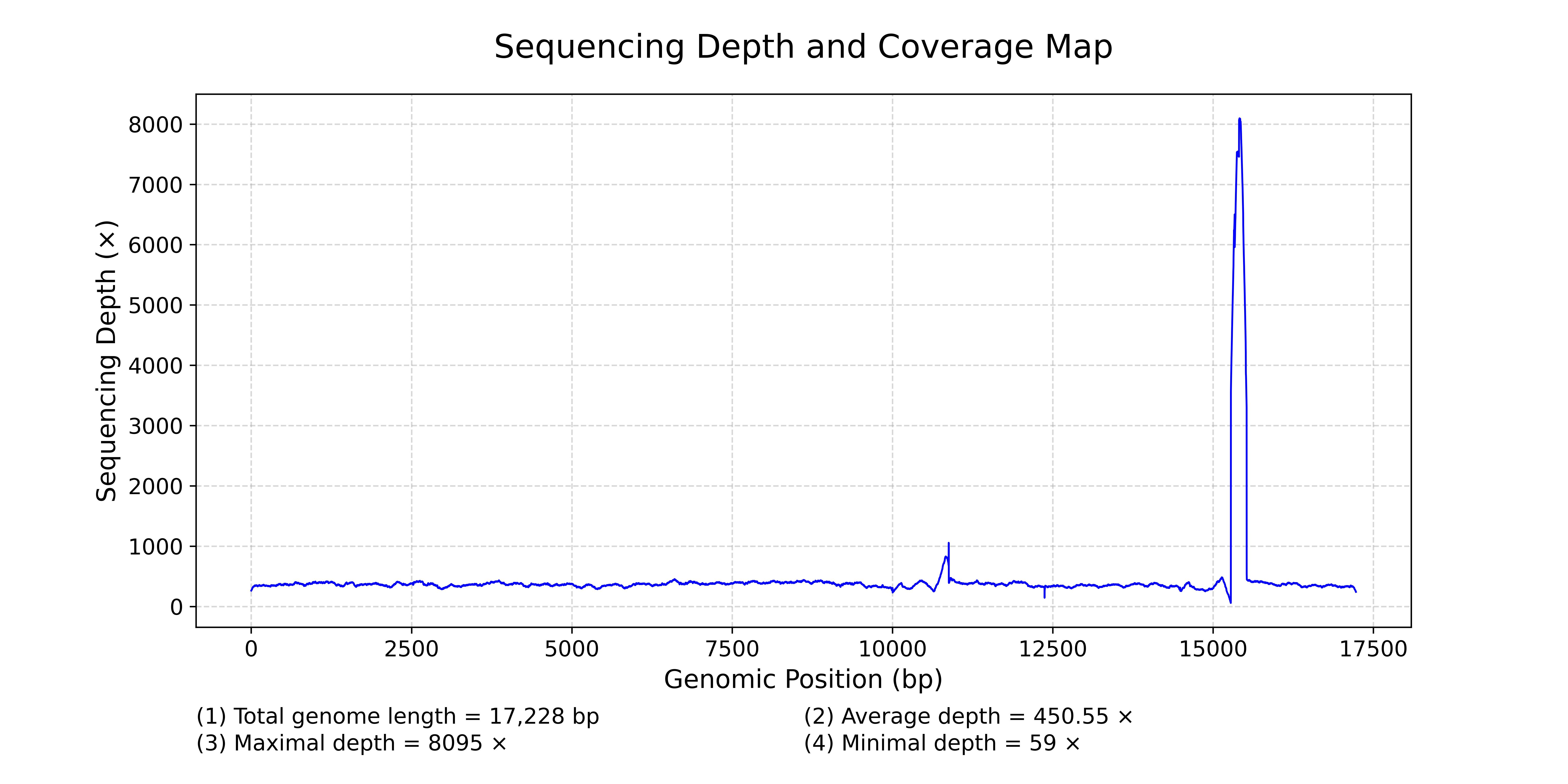

Supplement: Supplemental Material [file TMDN_A_2419423_SM8781.jpg]
