# Supplementary material for: Characterization and phylogenetic analysis of the complete mitochondrial genome of Aipysurus eydouxii Gray 1849 (Elapidae: Hydrophiinae)
Source: Mitochondrial DNA B Resour. 2024 Oct 25;9(10):1450–4. doi: 10.1080/23802359.2024.2419423 (PMC11514406; doi:10.1080/23802359.2024.2419423)
Supplement: OGV result showed no complex genes.html [file TMDN_A_2419423_SM8995.html]

123.fas.2.7.7.80.10.50.500.1.html  

```
Tandem Repeats Finder Program written by:
```

```
Gary Benson  
Program in Bioinformatics  
Boston University  
Version 4.09  

Please cite:
G. Benson,
"Tandem repeats finder: a program to analyze DNA sequences"
Nucleic Acid Research(1999)
Vol. 27, No. 2, pp. 573-580.

Sequence: NC_062614
Parameters: 2 7 7 80 10 50 500
Length:  17228
```

```
Tables:   1   

This is table  1  of  1  ( 1 repeats found )
```

```
Click on indices to view alignment
```

Table Explanation  
  

|  |  |  |  |  |  |  |  |  |  |  |  |
| --- | --- | --- | --- | --- | --- | --- | --- | --- | --- | --- | --- |
| Indices | Period Size | Copy Number | Consensus Size | Percent Matches | Percent Indels | Score | A | C | G | T | Entropy (0-2) |
| 14832--15508 | 200 | 3.4 | 200 | 98 | 0 | 1288 | 37 | 14 | 5 | 42 | 1.68 |

```
Tables:   1
```

The End!
